# Characteristics of reduced‐fat mayonnaise prepared by oleaster as a fat replacer and natural antioxidant

**DOI:** 10.1002/fsn3.3318

**Published:** 2023-05-07

**Authors:** Zahra Roshandel, Rezvan Zibaei, Khadije Abdolmaleki

**Affiliations:** ^1^ Student Research Committee, Department of Food Science and Technology, School of Nutrition Sciences and Food Technology Kermanshah University of Medical Sciences Kermanshah Iran; ^2^ Research Center of Oils and Fats Kermanshah University of Medical Sciences Kermanshah Iran

**Keywords:** fat substitute, low‐fat mayonnaise, natural preservative, rheological properties, stability

## Abstract

Due to the disadvantages of consuming fat and synthetic preservatives, the demand to reduce them in lipid‐based products like mayonnaise is increasing. In the current research, there were two goals, the first one was studying the effect of using oleaster flour in different concentrations (4%, 6%, and 8%) as a natural preservative, whereas the second one was studying the effect of oleaster as different fat replacement (FR) levels (10%, 20%, 30%, and 40%) on the physicochemical, antioxidant, and the rheological properties and stability of reduced‐fat mayonnaise samples. Given results showed that with increasing the oleaster concentration, the antioxidant property increased significantly. The peroxide value after 60 days of storage for the 30% FR 8 was 2.01%, compared to the control sample without antioxidant and with TBHQ, which were 10% and 2.68%, respectively. The highest stability index (100%) was observed in the 30% FR and 40% FR samples. In terms of rheological characteristics, the 30% FR 8 oleaster showed the highest viscosity and the lowest frequency dependency. It can be concluded that oleaster has a high potential to be used in the formulation of low‐fat mayonnaise as a fat replacer.

## Graphical abstract

In response to high‐fat related health concerns like obesity, diabetes, cardiovascular diseases, and cancer, many food scientists and technologists have aimed at developing low‐calorie food products. Noteworthy, once the fat is eliminated, some unfavorable changes in many sensory attributes of reduced‐fat foods, such as appearance, flavor, mouthfeel, and texture occur, which have restricted consumer acceptance and commercial success rate. So, In the current research, the impact of oleaster at different concentrations (4%, 6%, and 8% w/w), as a fat replacer, on the physical, textural and rheological characteristics and stability of reduced‐fat mayonnaise (10%, 20%, 30% and 40% fat reduction) was examined.

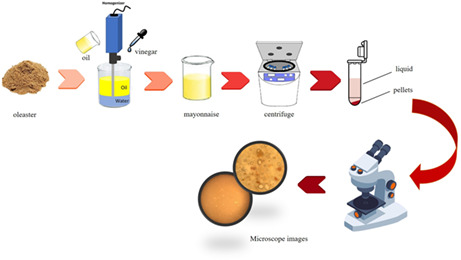

## 1 INTRODUCTION

Mayonnaise is the most high‐calorie and most popular food product as an oil‐in‐water emulsion in low pH, which contains 70%–80% oil (dispersed phase), vinegar (continuous phase), and egg yolk (Elsebaie et al., 2022). Among foods containing high amounts of oil, mayonnaise is susceptible to spoilage due to the autoxidation of unsaturated fats (Chatterjee & Bhattacharjee, 2015). Lipid oxidation in mayonnaise leads to the production of toxic compounds, which in turn cause an unpleasant taste and reduce the shelf life of products. The oxidation of unsaturated fatty acids is the main cause of chemical instability in emulsions. In order to inhibit lipid oxidation in mayonnaise, the use of antioxidants is necessary. For this purpose, synthetic antioxidants including butyl hydroxy‐toluene (BHT), butylate hydroxy anisole (BHA), and tri‐butyl hydroquinone (TBHQ) are widely used in mayonnaise (Kwon et al., 2015). Due to the toxic and carcinogenic effects of these compounds in high concentrations and the high demand for mayonnaise, the replacement of these chemical compounds with natural antioxidants should be considered. The addition of natural compounds to food products has a high potential to improve oxidative stability and health effects and therefore will be attractive to consumers. Also, by replacing these ingredients, mayonnaise producers will achieve the two goals of using natural ingredients and improving health (Gorji et al., 2016). In addition, in recent decades, changes in human lifestyles and dietary patterns have led to an increase in obesity and chronic cardiovascular disease. Therefore, the general policy of the food industry in different countries is to produce fat‐free or low‐fat foods with a flavor similar to the natural product (Kouhanestani et al., 2019). Given the adverse effects of excessive fat consumption on health, the identification of a suitable fat substitute in the formulation of high‐calorie foods is inevitable (Park et al., 2020). Fat substitutes are very diverse and include protein‐based and carbohydrate‐based alternatives or a combination of both of them. Carbohydrate‐based fat substitutes are a group of compounds derived from cereals, legumes, and plants with digestible or indigestible carbohydrates (Agyei‐Amponsah et al., 2021). Proteins with unique structure and functional properties in particular gelatin and whey protein are frequently credited with being the basic idea of fat substitutes (Yashini et al., 2021). Foods such as flour due to their oil composition, and carbohydrate and protein content can be considered fat replacers.

Oleaster flour is one of the fat substitutes with antioxidant properties. Oleaster is a tree or shrub with the scientific name *Elaeagnus angustifolia* from the family Elaeagnaceae and the genus Elaeagnus, which is also commonly called Russian olive. This shrub is native to North Asia, Europe, and Iran. Oleaster fruit is oval, fleshy, in the shape and dimensions of an olive, red–orange, astringent, and consumable with a mild taste, and due to the flour structure and nutritional value, it is mainly used in the food and pharmaceutical industries (Chen et al., 2014). This sweet fruit contains significant amounts of flavonoids, terpenoids, and phenolic compounds, including 4‐hydroxybenzoic acid, phenolic acid, and caffeic acid, and has antioxidant properties due to its flavonoids (Karkar & Şahin, 2022). Also, owing to the presence of sugars such as glucose and fructose, it can greatly replace sucrose in mayonnaise formulations (Sarraf et al., 2017). In addition, oleaster fruit contains vitamins K and C and also contains palmitoleic, linoleic, oleic, and linolenic fatty acids and its core also contains linoleic fatty acids, phospholipids, glycolipids, and beta‐sitosterol. Also, oleaster flour and crust due to high fiber in addition to improving nutritional properties can lead to increased consistency and viscosity of the product (Öztürk et al., 2018). Recent pharmacological studies indicate the therapeutic properties of this plant and its fruit as an anti‐inflammatory and analgesic agent in patients with arthritis, gastric ulcer, nausea, vomiting, jaundice, asthma, and bloating (Hamidpour et al., 2017). Therefore, considering the numerous properties of oleaster fruit, the importance of public health, and the production of food products with high nutritional value and good quality properties, this study was conducted to investigate the possibility of using oleaster flour with crust as a fat substitute in the production of low‐fat mayonnaise and its effect on physicochemical, antioxidant, and rheological properties and stability of this low‐fat emulsion.

## 2 MATERIALS AND METHODS

### 2.1 Materials

Xanthan gum, guar gum, potassium sorbate, sodium benzoate, TBHQ, and DPPH (2,2‐diphenyl‐1‐picryl hydrazyl) were prepared from Merck. Oleaster fruit was purchased from the local market of Kermanshah, Iran. After separating the impurities, it was completely ground and sieved with 40 mesh. Also, all other ingredients (sunflower oil, salt, sugar, egg yolk powder, and vinegar) were purchased from a market in Kermanshah, Iran.

### 2.2 Sample preparation

The mayonnaise formulation was obtained by adjusting the percentages of ingredients from various studies after pretests. The control sample consisting of the following weight percentages was prepared from different materials: 70% sunflower oil, 18% water, 0.5% egg yolk powder, 5% vinegar, 5% sugar, 1.5% salt, 0.2% xanthan gum and guar gum, 0.1% potassium sorbate and sodium benzoate, and 0.1% TBHQ. Mayonnaise treatments were prepared by adding oleaster powder in three different concentrations (4%, 6%, and 8%) as a fat substitute with levels of 10%, 20%, 30%, and 40%. First, the egg yolk powder and other powdered ingredients were mixed with water for 30 s in a Direct Driven Stirrer (FTDS‐410). Then, sunflower oil and vinegar were gradually added to the aqueous phase and mixed for 5 min to form an emulsion. After preparation, the mayonnaise is placed in glass jars with lids and then labeled. Samples were stored for 2 months at 4°C to be investigated at four different times in the future. Then, the physicochemical, oxidation, rheological, emulsion stability, and microstructure properties of the samples were evaluated.

### 2.3 Proximate compositions of the oleaster

Oleaster flour was analyzed according to the AACC method (AACC, 2000). The moisture content of flour was according to AACC No: 44‐01, ash percentage according to AACC No: 01‐08 with electric oven (Amalgams, Iran) at 550°C, fat content by Soxhlet method in automatic fat extraction machine (Soxhletsox, 406) according to AACC No: 25‐25, and the protein according to AACC No: 12‐46 and by semiautomatic Kjeldahl nitrogen analyzer (Auto Analyzer 130 Tecator CO) including three stages of digestion, distillation, and titration, and protein coefficient of 6.25 (Kouhanestani et al., 2019).

### 2.4 Peroxide index

The peroxide index was determined by titration method and according to AOCS (2006) standard procedure. Five gram of the oil phase extracted from mayonnaise was poured into the Erlenmeyer flask and 15 mL of acetic acid solution and 10 mL of chloroform were added. Then, 1 mL of saturated potassium iodine solution was added to the initial mixture and after being placed in a dark place for 1 min and adding 25 mL of distilled water and starch reagent, it was titrated with 0.1 N sodium thiosulfate and the volume used was recorded. The peroxide index was analyzed by using the following equation.

$$\mathrm{pV}\;\left(\mathrm{meq}\;O_{2}/\mathrm{kg}\;\text{oil}\right)=\frac{\left(\;S-B\;\right)\times N\times 1000}{W}$$

where *S* is the sample using thiosulfate volume, *B* is the blank using thiosulfate volume, *N* is the normality of NAOH, and *W* is the weight of the sample taken (Alizadeh et al., 2019).

### 2.5 Antioxidant activity

The free radical scavenging method was used to determine the antioxidant activity of the samples. For this purpose, 5 mL of the samples was mixed with 25 mL of methanol solution (75%) and stirred for 30 min, and then completely dissolved by ultrasonic (Memmert). The resulting mixture was centrifuged at 125 rpm for 10 min. Then, 1 mL of the supernatant of the centrifuged solution of each sample was mixed with 4 mL of 0.1 N DPPH solution (2 and 2 diphenyl‐1‐picryl hydrazyl), and it was kept in the dark for 30 min. The absorbance of each was measured at 517 nm using a spectrophotometer (UV–Vis Spectrophotometer, UNICO 2100). Finally, DPPH radical scavenging activity was calculated according to the following equation (Çakmakçı et al., 2015):

$$I\%=\frac{\left(\;\mathrm{AB}-\mathrm{AC}\;\right)\times 100}{\mathrm{AB}}$$

where AB and AC are the optical absorption of blank and investigated samples, respectively.

### 2.6 Rheological property measurements

#### 2.6.1 Steady‐state measurements

Rheological tests of mayonnaise samples were conducted (Anton Paar Rheometer, MCR 301) using parallel plate geometry (diameter = 40 mm, gap = 1 mm). The rheometer had a water circulator system to control temperature and all experiments were done at a constant temperature (25°C). For temperature equilibration, all samples were put on the lower plate for about 5 min before starting the tests. The steady‐state measurement of the samples was done in the shear rate range of 0.01–500 s^−1^. To check the flow curve of mayonnaise, rheological data were fitted to the power law model and Hershel–Bulkley model. Hershel–Bulkley model with *R* ^2^ higher than .95 is the best model to describe the flow behavior of the sample.

$$\tau =k{\left(\gamma^{{}^{\circ}}\right)}^{n}+\tau_{0}$$

where *k* is the consistency factor (Pa s^*n*^), $\gamma^{{}^{\circ}}\;$ is the shear rate (s^−1^), *τ* is the shear stress (Pa), $\tau_{0}$ is the yield stress, and *n* is the flow behavior index (unitless).

#### 2.6.2 Dynamic rheological measurements

A strain sweep was applied from 0.005% to 1000% at the constant frequency of 1 Hz to determine the linear viscoelastic region (LVR). The frequency sweep was performed to measure the viscoelastic parameters in a frequency range of 0.1–100 Hz at a constant strain of 1% (under LVR). The elastic modulus (*G*′, Pa) of the samples as a function of frequency was characterized using the following equation (Chatterjee & Bhattacharjee, 2015):

$$G^{\prime }=A{\left(\omega \right)}^{b}$$

where *A* is structure strength (Pa.s rad^−1^), *b* is the gel structure type (unitless), and *ω* is the angular frequency (Hz).

### 2.7 Emulsion stability

To measure the stability of mayonnaise samples, 10 g of the sample (*F*_0_) was transferred into 15 mL plastic tubes. The tubes were then heated at 60°C for 30 min in a water bath (Memmert) and centrifuged (UNIVERSAL320/320R) at 1792 *g* for 10 min. The emulsion stability was then obtained from the following equation (Sahan et al., 2015):

$$\text{Emulsion stability}\%=\frac{F_{1}\times 100}{F_{0}}$$

where *F*_1_ is the weight of the emulsified phase.

### 2.8 Microscopic analysis

Microscopic images were taken using an optical microscope (Olympus). A small amount of the emulsion sample was placed on the slide and covered with a cover sheet to ensure that no air was trapped between the cover and the slide. After 5 min of equilibration, the emulsion micrograph was taken at a magnification of 1000. Images were selected and reported from five replicates (Javidi et al., 2019).

### 2.9 Statistical analysis

One‐way analysis of variance (ANOVA) and Duncan's multiple‐range tests were used to compare differences among mean values of mayonnaise properties. Mean and standard deviation were reported and the significance was stated at *p* ≤ .05.

## 3 RESULTS AND DISCUSSION

### 3.1 Characterization of the oleaster flour

The amounts of ash, moisture, fat, and protein were 1.98%, 8.66%, 1.19%, and 6.43%, respectively. The results showed that oleaster flour is a good source of protein and in this regard has a high nutritional value. In a study conducted by Sahan et al. (2015) in Turkey, the moisture, protein, and fiber content of peeled oleaster flour samples from two different genotypes were reported to be 18.99–19.78 g/100 g, 3.74–4.51 g/100 g, and 20.67–23.55 g/100 g, respectively. The amounts of chemical compounds in oleaster flour in the present study were relatively similar to other studies and slight differences can be attributed to differences in oleaster species, and climatic and geographical conditions (Sahan et al., 2015).

### 3.2 Peroxide index

In mayonnaise, which is an oil‐in‐water emulsion consisting of small droplets of fat dispersed in the aqueous phase, lipid oxidation occurs at the interface of oil‐in‐water particles. Oxidation products lead to an unpleasant taste and odor and changes in the color and texture of the food, thereby reducing the quality and shelf life of the food. The peroxide index is used to measure the primary oxidation products (peroxides and hydroperoxides) that indicate the onset of oxidation and deterioration (Ghorbani Gorji et al., 2019; Khalid et al., 2021). The peroxide values of the different samples are compared in Table 1. As can be seen, the values of the peroxide index on the first day of production (immediately after production) in all samples are equal and in fact the same peroxide index of sunflower oil was used in the formulation. The peroxide index in all samples increased during storage and after 60 days. As can be seen, the rate of increase in peroxide number during 60 days of storage in samples with 4%, 6%, and 8% of oleaster was lower than the control sample and the sample with TBHQ as a synthetic antioxidant. These results can show the positive and effective role of oleaster in the oxidative stability of mayonnaise. The results of many studies show that oleaster, due to its high phenolic content, inhibits lipid oxidation and delays the increase in oxidation products in the food. Phenolic compounds, due to their structure and hydroxyl group in their structure, can eliminate reactive oxygen species and are antioxidants. Various studies have shown a direct relationship between phenolic content and the ability to scavenge free radicals and thus inhibit oxidation (Gorji et al., 2016; Martillanes et al., 2020). According to the results of the study done by Sahan et al. (2019) which used oleaster flour to prepare a type of cookie, a significant increase in the total phenol content was observed in samples enriched with oleaster flour compared to the control sample. Also, the amount of total phenol in the oleaster flour cookie sample with crust was 79% higher than the one without crust and these samples had a much higher antioxidant capacity than the control sample (Sahan et al., 2019). Also, as the results of stability analysis show, the addition of oleaster to the formulation has improved the emulsion stability. In the study of Sahan et al. (2015), similar results were obtained and it was observed that oleaster flour can significantly improve emulsion capacity and emulsion stability. This increase in the stability and uniformity of the structure in mayonnaises with oleaster leads to a decrease in porosity and an increase in texture density. In fact, reducing porosity due to the reduction of oxygen available as an oxidation substrate can be effective in reducing oxidation. Therefore, it can be concluded that in samples with oleaster, a significant increase in phenolic content and a decrease in the amount of oil and oxygen as oxidation substrates has led to oxidative stability in these samples compared to the control sample and even samples with TBHQ.

**TABLE 1:** Peroxide index of mayonnaise samples during cold storage.

Sample (%30FR)	0 days	15 days	30 days	60 days
4	1.16 ± 0.00^Da^	1.73 ± 0.02^Cc^	1.94 ± 0.05^Bc^	2.5 ± 0.02^Ac^
6	1.17 ± 0.02^Da^	1.40 ± 0.05^Ce^	1.81 ± 0.04^Bd^	1.91 ± 0.05^Ae^
8	1.16 ± 0.01^Da^	1.63 ± 0.06^Cd^	1.90 ± 0.03^Bc^	2.01 ± 0.02^Ad^
Control	1.18 ± 0.02^Da^	2.78 ± 0.10^Ca^	3.39 ± 0.03^Ba^	10.35 ± 0.05^Aa^
Control (TBHQ)	1.16 ± 0.01^Da^	1.92 ± 0.08^Cb^	2.60 ± 0.06^Bb^	2.68 ± 0.03^Ab^

*Note*: Results are reported as the mean ± standard deviation (*n* = 3). Capital letters display differences between the storage times and small letters show the differences between the mayonnaise samples.

### 3.3 Antioxidant activity

The dangerous side effects of many synthetic antioxidants and the extraordinary benefits of natural antioxidants have led to a growing interest in the use of antioxidants with natural resources in diets. Natural active compounds, such as phenolic compounds found in the fruits, flowers, seeds, and leaves of many plants, have high antioxidant activity because they can inhibit and scavenge free radicals (Hamidpour et al., 2017). In the present study, to evaluate the antioxidant activity of oleaster flour, the free radical scavenging method of DPPH was used and the antioxidant power of oleaster was compared with the TBHQ. The results of the antioxidant activity of different samples are shown in Table 2. As the results show, the antioxidant activity of the sample with TBHQ (control sample) was significantly higher than the samples with oleaster. However, the lowest antioxidant activity observed in samples with 4% oleaster in 30% replacement (62.87%) was only 33% less than the control sample (95.14%). As can be seen, with increasing the amount of oleaster from 4% to 8% in 30% replacement, antioxidant activity has significantly increased. In a study that used oleaster flour to enrich yogurt, similar results were obtained. In this study, it was observed that the antioxidant activity of the samples increased with increasing the amount of oleaster flour, and the ability to inhibit DPPH free radicals in samples with a crust was higher than in samples without a crust. Because the phenolic content in the oleaster crust is higher and has polyphenols such as coniferyl alcohol and (E) isoeugenol (Öztürk et al., 2018), the results of many studies show the high antioxidant activity of oleaster. In a study that used oleaster flour in the production of fruit ice cream, the antioxidant activity of oleaster was investigated using several different methods, including the free radical scavenging method (DPPH) and ferric reducing antioxidant power (FRAP). In the FRAP method, the results showed that the reducing power of oleaster flour was close to Trolox. Also, in the DPPH method, the results showed that the scavenging capability of oleaster flour was higher than that of BHA and BHT and close to the antioxidant power of α‐tocopherol. Finally, it was reported in this study that due to the acceptable and desirable antioxidant power of oleaster flour and crust, it can be used as a source of natural antioxidants (Çakmakçı et al., 2015). In another study, polysaccharides in oleaster pulp were extracted and their structure and antioxidant activity were investigated. The results showed that these polysaccharides have high antioxidant activity and can be used as natural antioxidant sources (Chen et al., 2014). As can be seen in Table 2, the antioxidant activity of samples with a constant amount of oleaster (8%) decreased with increasing the replacement percentage from 10% to 40%. The cause of this phenomenon can be explained by the fact that with increasing replacement percentage, the amount of oil has decreased and the amount of water has increased. It can be said that the hydroxyl groups in oleaster are involved in bonding and interacting with water molecules and as a result, are out of reach and this factor has led to a decrease in antioxidant activity at higher replacement percentages.

**TABLE 2:** Antioxidant activity of mayonnaise samples.

Sample	Inhibition (%)
Full fat 0	95.14 ± 0.5^a^
10% FR 8	86.2 ± 1.5^b^
20% FR 8	81.49 ± 2.0^c^
30% FR 4	62.87 ± 1.2^f^
30% FR 6	67.25 ± 1.9^e^
30% FR 8	78.04 ± 2.0^c^
40% FR 8	75.01 ± 1.0^d^

*Note*: Different small letters indicate significant difference (*p* ≤ .05) between treatments.

### 3.4 Rheological properties

#### 3.4.1 The steady shear properties

The effect of oleaster replacement on the viscosity of mayonnaise in different shear rates was investigated. The viscosity of all samples decreased with increasing shear rate and it confirmed the pseudoplastic behavior of mayonnaise. This behavior in concentrated emulsions like mayonnaise is attributed to structural breakdown and deformation of flocs under shear which result in the apparent viscosity reduction. Similar results have been reported in mayonnaise by Liu et al. ( 2007, 2018), where a droplet–droplet interaction breakdown with increased shear rate has been noted. Apparent viscosity (*μ*) in the shear rate of 0.1, consistency index (*k*), yield stress (*τ*_0_), and flow behavior index (*n*) of mayonnaise were calculated using the Herschel–Bulkley fitting and the findings are presented in Table 3. The viscosity of samples with a constant amount of oleaster (8%) increased with increasing the replacement percentage from 20% to 30%, but with further replacement, the viscosity decreased. The reduction of viscosity may be related to the higher water content in a sample. These results were in agreement with the findings of Werlang et al. (2021). In addition, as shown in Table 3, the Hershel–Bulkley equation is the best model to compare different samples due to its high coefficient determination (*R* ^2^). The flow index (*n*) of samples was concluded in the range of 0.23–0.41. The amount of flow behavior index (<1) shows the shear thinning (pseudoplastic) behavior of mayonnaise. The highest consistency index was observed in the sample containing 8% oleaster with 30% replacement which was clearly higher than that of other mayonnaise samples. The consistency index of 20% FR and 8% oleaster was closer to the control compared with other samples. Also, it was observed that the consistency index increased as oleaster concentration increased. The yield stress shows the solid‐to‐liquid state transition. There was a significant difference (*p* < .05) between the yield stress values for all mayonnaise samples and this factor was within a range of 10.04–196.72 Pa. However, a sample of 40% FR showed the lowest consistency index and yield stress value. In the research conducted by Tekin and Karasu (2020) and Li et al. (2012), similar results were obtained. They stated that oil concentration in mayonnaise plays a key role to improve the rheological characteristics, and higher oil content leads to a higher consistency index and yield stress (Li et al., 2012; Tekin & Karasu, 2020). In terms of rheological aspects, a suitable fat substitute in mayonnaise should have several factors as follows: high density and viscosity, pseudoplastic flow behavior, and sufficient yield stress (Heydari et al., 2021). Therefore, the sample containing 8% oleaster with 30% FR can be introduced as a suitable fat replacer.

**TABLE 3:** The Hershel–Bulkley equation parameters and *η*_0.1_ for the mayonnaise samples after 1 day of storage.

Sample	$\tau_{0}$ (Pa)	*K* (Pa.s)	*n*	*η* (Pa.s)
Full fat 0	173.30 ± 2.01^b^	52.20 ± 2.05^c^	0.31 ± 0.02^c^	1795 ± 5^b^
20% FR 8	112.44 ± 2.10^c^	56.32 ± 1.65^b^	0.30 ± 0.02^cd^	1661 ± 11^c^
30% FR 4	18.31 ± 3.12^d^	26.07 ± 2.44^e^	0.46 ± 0.01^a^	636 ± 8^e^
30% FR 6	114.00 ± 1.87^c^	36.14 ± 3.09^d^	0.28 ± 0.02^d^	1284 ± 10^d^
30% FR 8	196.72 ± 3.30^a^	100.26 ± 1.80^a^	0.23 ± 0.02^e^	2005 ± 6^a^
40% FR 8	10.04 ± 2.66^e^	10.00 ± 1.38^f^	0.41 ± 0.03^b^	292 ± 5^f^

*Note*: Different small letters indicate a significant difference (*p* ≤ .05) between treatments.

#### 3.4.2 Dynamic viscoelastic properties

**FIGURE 1:**
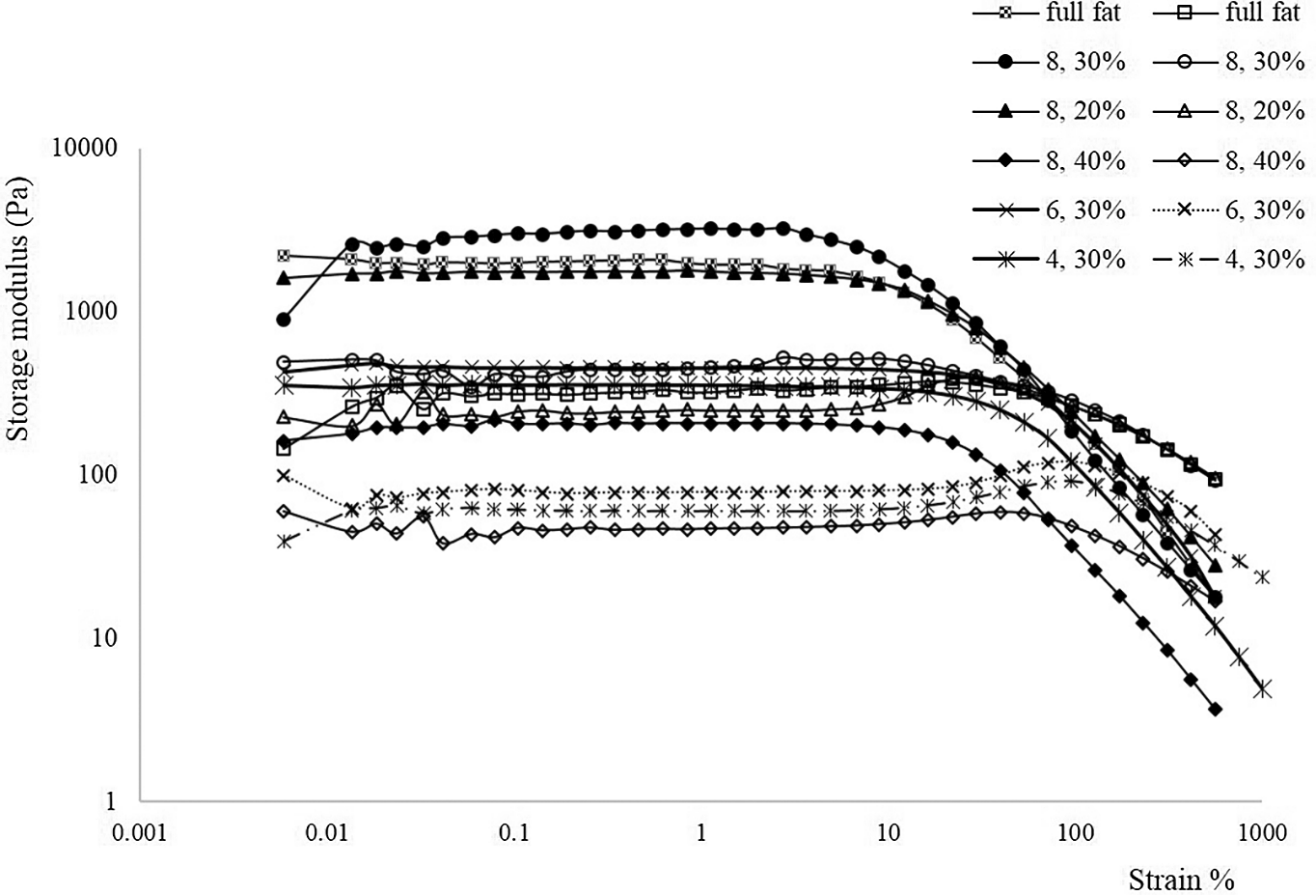
Strain sweep test of the mayonnaise samples.

A small‐amplitude oscillatory measurement should be carried out within the linear viscoelastic (LVE) region to minimize the destruction of the mayonnaise sample during the dynamic test. In all mayonnaise samples, *G*' starts to decrease after the LVE range showing structural breakdown while *G*" exhibited an overshoot in the nonlinear region which has been characterized as type III (weak strain overshoot) behavior. This phenomenon has been reported in concentrated emulsions like mayonnaise (Anvari & Joyner, 2018). Figure 1 shows the result of the strain sweep test at a constant frequency of 1 Hz. For all samples, the storage modulus (*G*') was higher than the loss modulus (*G*") in LVE (linear viscoelastic) region which shows elastic‐dominated behavior. The highest *G*' value (3100 Pa) belongs to the sample 30% FR‐containing 8% oleaster. Furthermore, all mayonnaise samples were classified into gel‐like emulsions because the storage modulus was higher than the loss modulus in all tested frequency ranges and the crossover between the two moduli did not show (Mancini et al., 2002). Also, an increase in frequency caused a slow increase in *G*' and *G*" showing mayonnaise had low time dependency. Laca et al. (2010) and Liu et al. (2018) have reported similar behavior in mayonnaise. The *G*' was modeled as a power function of angular frequency and *A* and *b* values were reported as intercepts and slopes, respectively, in Table 4. As can be seen, with increasing the amount of oleaster from 4% to 8% in 30% FR, the *A* value has significantly increased which means there are effective interactions between oil droplets. Similar results were previously found when cornstarch nanocrystals were used as a fat replacer in a reduced‐fat O/W emulsions matrix (Javidi et al., 2019). The *A* value of samples with a constant amount of oleaster (8%) increased with increasing the replacement percentage from 20% to 30%. This can be related to the ability of oleaster as the fat replacer to strengthen the fat‐reduced mayonnaise structure. It is believed that the mayonnaise with a higher oil content would display a greater *A* value due to the interaction of fat globules among each another and closely packed oil droplets. As mentioned, this funding was not obtained for the samples with 30% FR. Therefore, it may be concluded that the oleaster probably was capable of developing hydrogen bonds with water molecules, so the strong structure formed was more efficient than close‐packed particles, resulting in higher oil content (Carcelli et al., 2020; Chang et al., 2017). However, the *A* value decreased in 40% FR and the structured network was weaker than other samples probably because of a lower oil concentration in the dispersed phase. As seen in Table 4, the values of b as the frequency dependence parameter were in the range 0.08–0.13, which were close to the magnitudes of true gel (*b* = 0) and much lower than those stated for a Maxwellian system (*b* = 2). Heydari et al. (2021) also reported the same results for power function parameters in reduced‐fat O/W emulsions.

**TABLE 4:** Frequency dependence of the elastic modulus for the mayonnaise samples.

Sample	*A* (Pa.s rad^−1^)	*b*	*R* ^2^
Full fat 0	1563 ± 7.8^b^	0.08 ± 0.01^b^	.95
20% FR 8	1382.70 ± 9.6^c^	0.09 ± 0.00^b^	.98
30% FR 4	296.11 ± 8.1^d^	0.13 ± 0.01^a^	.96
30% FR 6	356.00 ± 3.4^c^	0.13 ± 0.00^a^	.95
30% FR 8	1788.5 ± 5.5^a^	0.08 ± 0.01^b^	.99
40% FR 8	158.6 ± 4.2^e^	0.12 ± 0.01^a^	.96

*Note*: Different small letters indicate a significant difference (*p* ≤ .05) between treatments.

### 3.5 Emulsion stability

Stability is a very important property in mayonnaise as an oil‐in‐water emulsion. Factors affecting the stability of emulsions include viscosity, the size distribution of fat globules, concentration, and type of emulsifiers (Lee et al., 2013). The stability index of the control sample (without oleaster) and also the FR samples containing oleaster at different levels (4%, 6%, and 8%) are listed in Table 5. The results showed that the stability in all samples increased significantly with an increasing replacement percentage from 10% to 40% (*p* < .05). The sample of mayonnaise containing 6% oleaster with 10% replacement has the lowest stability (49.58%) and in all samples containing oleaster (4%, 6%, and 8%) with 30% and 40% replacements, the highest stability (% 100) was observed. These results were consistent with the results of Abdul Aziz et al. (2012). They found that the emulsifying and stabilizing agents in mango peel and paste were carbohydrate polymers such as pectin, which may increase the viscosity of the aqueous phase and reduce the tendency of fat globules to move and coalesce (Abdul Aziz et al., 2012). Mechanisms such as creaming, flocculation, coalescence, and phase inversion destabilize the emulsion system (Lee et al., 2013). Creaming is a process in which emulsion droplets separate from the continuous phase and tend to migrate up or down depending on the density difference between the continuous phase and the dispersed phase (Javidi et al., 2019). In high‐fat foods when the fat content is reduced to increase viscosity and reduce the speed of droplets, it is necessary to add some thickeners to the aqueous phase, in which fat substitutes play an important role (Lee et al., 2013). According to Park et al. (2020), the low stability of high‐fat mayonnaise may be due to the incorporation of oil droplets due to insufficient stabilizers (Park et al., 2020). As can be seen in Table 5, with increasing the amount of oleaster from 4 to 8 in the 20% replacement compared to the 10% replacement and even the control sample, the stability increased significantly (*p* < .05). This is because the higher the initial particle content (amount of oleaster), the stronger the regular structure of the emulsion molecules, the delayed phase separation, and the consequent increase in stability. Our result was in line with the finding obtained by Werlang et al. (2021) who reported that with an increase in the concentration of native and annealed oat starches in mayonnaise, the stability was increased (Werlang et al., 2021). The significant effect of modified starch with 30% and 50% fat replacement (FR) on the physical stability of mayonnaise has been investigated by Park et al. (2020). Their results showed that by increasing the FR ratio from 30% to 50%, the stability of the samples increased (Park et al., 2020). Sahan et al. (2015) evaluated the properties of oleaster flour. They found that the increase in stability values was probably due to the presence of high dietary fiber in the oleaster crust. Oleaster flour acts as a bulk barrier between oil droplets and slows down the movement of oil droplets by increasing the viscosity of the aqueous phase, thereby preventing the droplets from merging and creaming (Sahan et al., 2015). Therefore, it can be concluded that in all samples containing oleaster (4%, 6%, and 8%) with 30% and 40% FR, the stability is higher than the control sample and it seems that oleaster flour has an improving effect on emulsion stability.

**TABLE 5:** Emulsion stability of the mayonnaise samples.

Sample	Stability (%)
Full fat 0	80.68 ± 4.0^e^
10% FR 4	78.27 ± 2.0^e^
10% FR 6	49.58 ± 2.0^g^
10% FR 8	57.16 ± 2.0^f^
20% FR 4	93.1 ± 1.0^c^
20% FR 6	87.50 ± 1.0^d^
20% FR 8	97.18 ± 0.8^b^
30% FR 4	100.00 ± 0.0^a^
30% FR 6	100.00 ± 0.0^a^
30% FR 8	100.00 ± 0.0^a^
40% FR 4	100.00 ± 0.0^a^
40% FR 6	100.00 ± 0.0^a^
40% FR 8	100.00 ± 0.0^a^

*Note*: Different small letters indicate a significant difference (*p* ≤ .05) between treatments.

### 3.6 Microstructure

**FIGURE 2:**
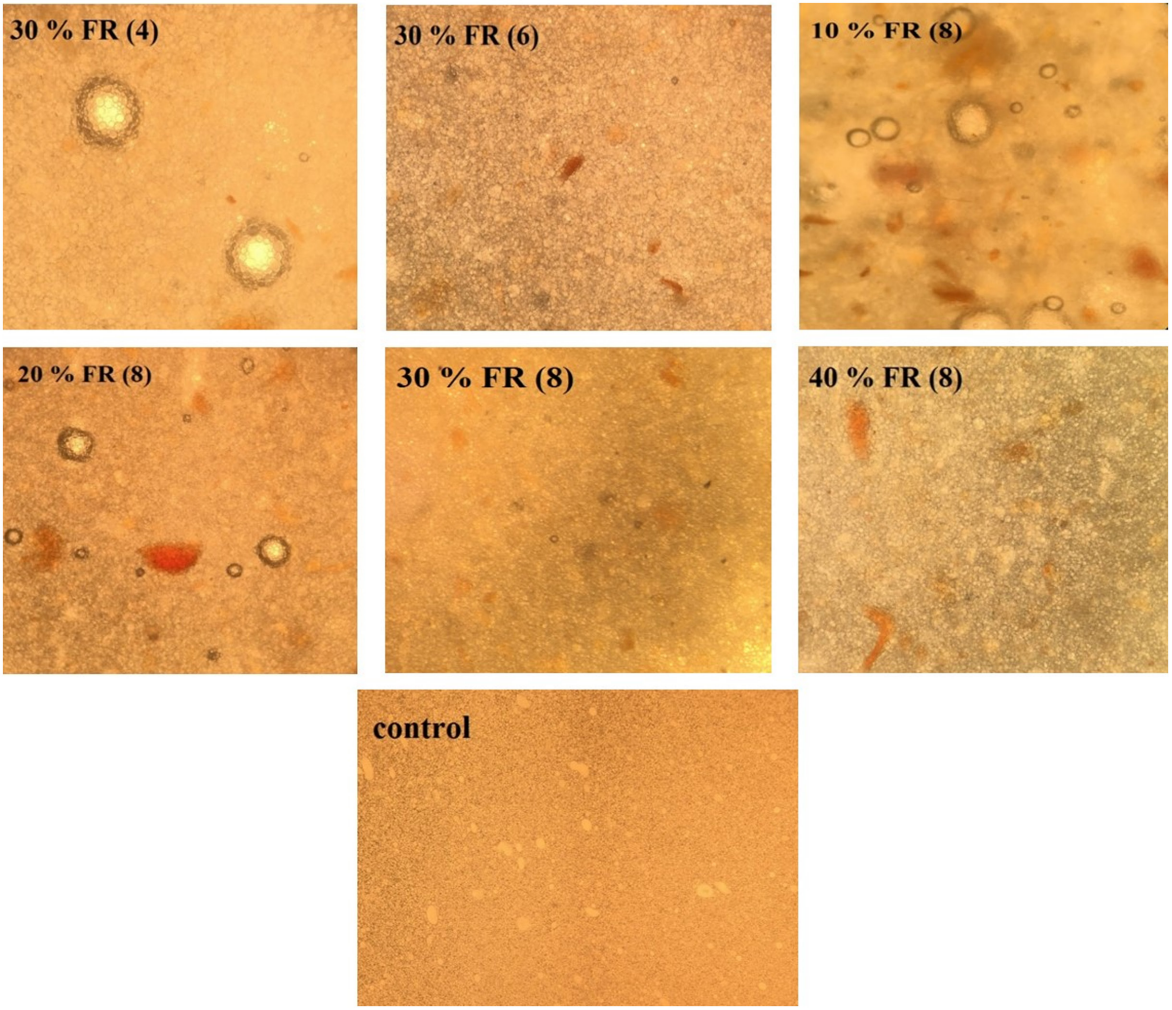
Optical microscopy images of the mayonnaise samples.

To evaluate the mayonnaise microstructure, optical microscopy has been used which contains oil molecules dispersed in a water phase. Microscopic images of the samples were shown in Figure 2. As can be seen, with increasing the amount of oleaster from 4% to 8% in 30% replacement, the uniformity of the emulsion structure and the dispersion of oil particles in water have increased. Flour oleaster seems to maintain the structure of the emulsion and its higher stability. In a study that examined the functional properties of oleaster flour, the results of the emulsifying capacity study show that oleaster flour can effectively enhance and improve the emulsifying capacity of albumin. Oleaster flour has also been able to significantly increase the stability of the emulsion. Therefore, it can cause more uniformity in the emulsion structure (Sahan et al., 2019). In samples with an equal amount of oleaster (8%) in 10% and 20% replacement, larger droplets and more particle flocculation are seen, while in higher replacement percentages (30% and 40%), the structure is more uniform and particle flocculation is less. These results are according to the results obtained from the stability test and the stability of the samples at 30% and 40% replacement was much more desirable.

## 4 CONCLUSIONS

In this research, the effect of oleaster as a fat replacer and natural antioxidant in mayonnaise was investigated. The results showed that the antioxidant activity increased significantly by increasing the amount of oleaster from 4% to 8% in 30% substitution. The peroxide value in the samples with oleaster was lower than the control sample and the sample with TBHQ after 60 days of storage. Also, due to the presence of high fiber, oleaster flour acts as a stabilizer and bulky barrier, which, in addition to increasing the viscosity, prevents the movement of the oil droplets and thereby stabilizes the emulsion. As a result, oleaster flour, especially at higher levels and 30% FR, created samples with a homogeneous structure and good stability, high antioxidant, and suitable viscoelastic properties. Therefore, it seems that oleaster can be used as a suitable fat replacer in food products. Moreover, due to the fact that oleaster contains sugar and mayonnaise samples formulated with it were sweeter, with this replacement, the amount of sugar can be also reduced in mayonnaise. It is suggested that the use of oleaster as a sugar substitute needs to be investigated in the future.

## CONFLICT OF INTEREST STATEMENT

The authors declare that they have no conflict of interest.

## ETHICS STATEMENT

This study does not contain any human or animal testing.

## Data Availability

The data that validate the results of this research are available from the corresponding author upon reasonable request.
